# A RCT Comparing Specific Intensive Cognitive Training to Aspecific Psychological Intervention in RRMS: The SMICT Study

**DOI:** 10.3389/fneur.2014.00278

**Published:** 2015-01-13

**Authors:** Flavia Mattioli, Chiara Stampatori, Fabio Bellomi, Maura Danni, Laura Compagnucci, Antonio Uccelli, Matteo Pardini, Giuseppe Santuccio, Giuditta Fregonese, Marianna Pattini, Beatrice Allegri, Raffaella Clerici, Annalisa Lattuada, Cristina Montomoli, Barbara Corso, Ruggero Capra

**Affiliations:** ^1^Neuropsychology Unit, Spedali Civili di Brescia, Brescia, Italy; ^2^Clinica Neurologica, University of Ancona, Ancona, Italy; ^3^Clinica Neurologica, MS Center, University of Genoa, Genoa, Italy; ^4^Neurology Unit, Azienda Ospedaliera Valtellina Valchiavenna, Sondrio, Italy; ^5^Neurology Unit, Fidenza Hospital, Parma, Italy; ^6^Neurology Unit, Como Hospital, Como, Italy; ^7^Biostatistics Unit, Department of Public Health, Experimental and Forensic Medicine, Pavia University, Pavia, Italy; ^8^MS Center, Spedali Civili di Brescia, Brescia, Italy

**Keywords:** multiple sclerosis, cognitive rehabilitation, attention, executive functions memory, information processing speed, multiple sclerosis cognitive rehabilitation

## Abstract

**Background:** Specific cognitive rehabilitation in multiple sclerosis (MS) resulted to be effective compared to no treatment. So far the possible role of an aspecific psychological intervention on cognition has not been investigated.

**Objective:** The aim of the SMICT RCT was to compare the efficacy of a specific cognitive training with an aspecific psychological intervention in relapsing-remitting MS patients.

**Methods:** From a sample of 150 patients, with the same disability and immunomodulatory therapy, submitted to neuropsychological examination, 45 impaired in at least one test were included and 41 randomized to have either a specific cognitive training for the impaired function (22) or to an aspecific psychological intervention (19) for 4 months, starting after baseline examination. Neuropsychological tests and functional scales were administered at baseline and 1 year later.

**Results:** After 1 year, the mean number of pathological tests was significantly lower in the specific treatment group, compared to the aspecific group. Memory and attention/speeded information processing functions were mostly improved. Depression and quality of life were not different between groups at follow up.

**Conclusion:** Our study demonstrates that an intensive and domain specific cognitive approach results to be more effective than aspecific psychological intervention in patients with MS.

## Introduction

Patients affected by multiple sclerosis (MS) often suffer from impairments in several cognitive domains (1). The incidence of depression has also been found to be higher in MS (2, 3). For these reasons, MS patients are more prone to loss of employment, reduced social and working abilities, and worsened quality of life (4, 5). The effects of immunomodulatory drugs on cognitive deterioration are not completely clarified (6). Indeed, cognitive interventions are worth investigating, since the methodological biases of the initial reports have been overcame by recent research, supporting the efficacy of cognitive training in MS. The heterogeneity in disease severity, the undetermined type of treatments and the inappropriateness of outcome measures were the most common confounders (7–9). However, the rehabilitation of selected cognitive domains (e.g., attention, executive functions, memory) has been found to improve trained function (10–14), with reported correlates of brain functional activations (15–19). Targeted treatments were compared with no treatment (12, 20) or control treatments (14, 21); though a possible aspecific due to a simple taking care effect has never ruled out so far.

The aim of the Sclerosi Multipla Intensive Cognitive Training (SMICT) trial is to verify the efficacy of a specific (S) intensive cognitive training for attention/speeded information processing (AIP), executive functions (EF), and memory (M) compared to an aspecific psychological intervention (A) – a placebo “psychological treatment” – in improving relapsing-remitting MS patients’ cognitive impairment over one year.

## Patients and Methods

Ten Italian MS Centers participated in the SMICT study, whose coordinator Center was the Neuropsychology Unit of Brescia Hospital, which was responsible for data collection in an accessible Data base and interpretation of the results. The randomized clinical trial was registered in the Spedali Civili of Brescia trial Register (NP:560).

Randomization (according to a computer-generated list of random number) and statistical analysis of data were carried out by an independent center, from which all the Centers received the patients’ number.

Patients diagnosed as affected with MS, according to Poser et al. criteria (22) with a relapsing remitting course were included in the study, after their signed informed consent was obtained. The study was performed according to the Helsinki Declaration and after the approval of the Ethical Committee (Comitato Etico Provinciale di Brescia, January 2010). Patients’ enrollment started on June 2010 and ended 31 December 2011.

To participate in the study, patients needed to have been prescribed interferon beta 1A 44 mcg three times/week no later than 6 months before, in order to have the most homogeneous drug regimen in patients. This first line therapeutic regimen was chosen, as it has been shown to be effective on several neuropsychological measures (6)Patients were included only if impaired (age corrected *z*-score ≤1.5 SD to norms) in at least one of the following test of the Italian version of the Rao’s Brief Repeatable Battery: Paced Auditory Serial Addition Task (PASAT 2″, PASAT 3″), Simbol Digit modality Test (SDMT), Spatial Recall Test (SPART) 10/36, and Delayed Recall (SPART D), Selective Reminding Test Long-Term storage (SRT LTS), Consistent Long-Term Retrieval (SRT CLTR), Delayed Recall (SRT DR) (23), Controlled Oral Words Association (COWA) with the Phoneme (P) and Category (C) modalities (12), and Stroop test (24). Eleven test’s scores were obtained for each evaluation plus three functional scale scores. Exclusion criteria were dementia (excluded by means of anamnestic reports as well as MMSE >24 in patients), previous or present psychiatric disorders (requiring pharmacological treatment) and clinically evident relapse in the previous 6 months.

To detect a reduction of 2 (SD = 1.5) in the number of pathological tests after the specific treatment, which is in agreement with our previous study, with a two-sided 5% significance level and a power of 90%, a sample size of 14 patients per group was necessary. Given an anticipated drop out of 30%, the total number of patients for each group increases to 18 (25).

The disease duration, the disability in the Expanded Disability Status Scale [EDSS, Kurtzke (26)], the relapse rate and steroid consumption (grams of intra venous methylprednisolone) in the previous year were registered.

The neuropsychological battery, the Modified Fatigue Impact Scale [MFIS, Kos et al. (27)] and MSQoL (28) were administered at baseline and after one year. Alternative forms of the neuropsychological tests were used, in order to avoid test retest effects and learning effects. As it was previously shown (12, 13) that immediate post training significant improvement in attention/executive function tests due to specific training is obtained and persists after 6 months, we decided to assess this persistence at 1 year. After inclusion, patients were randomly assigned to S treatment or A treatment, whose scheduled duration was 15 consecutive weeks with a frequency of two 60’ sessions per week, to be started in 2 weeks after baseline evaluation, according with standardized procedures, by an expert neuropsychologist, different from the evaluating one. An out patient regimen was used in all the centers; it was considered a maximum of three lost sessions as a cut off to be excluded from the study.

S treatment was administered according to the impaired neuropsychological function: Plan a Day software of the Rehacom (www.schuhfried.at) was used if a patient resulted impaired in EF (that is if his/her poor score was in the Stroop test or in the COWA P or COWA/C); Memory software of the same package was used if the patient was impaired in either the SRT or SPART verbal or spatial memory measures and the previously described (29)A/IP training, if he/she resulted impaired in AIP domain (pathological PASAT 2″, PASAT 3″, SDMT). If a patient was impaired in more than one domain, all the single domain trainings were balanced in the hourly session each time. Exercises complexity was adapted each time to the severity of each single patient’s impairment in the selected domain, with the aim that the exercise had to be challenging in each treatment session.

### Plan a day

The Plan a Day procedure trains the patient’s ability to organize, plan and develop solution strategies, employing realistic simulations of a set of scheduled dates and duties to be organized at specific places in a small city map. Times for planning and schedules are registered for each patient at each session and only improvement and acquisition of sufficient planning abilities for fulfilling all the appointments required led to an improved level in the following treatment session. Fifty four levels of increasing complexity are available, in order to challenge any grade of impairment. This was considered a strategic behavior acquisition. For further description of the treatment, see Mattioli et al. (12).

### Memory

Patients were asked to give answer to multiple choice or open questions about tales of increasing length, which were presented on the PC, whose complexity was chosen on the basis of the patient’s memory impairment. Ten levels of difficulty – also with interfering condition of two or three tales alternatively presented with the other tales’ questions – were progressively presented to the patients.

### A/IP training

A specific speeded information training with increasing velocity (from 4000 to 1800 ms interval), which has been shown to be effective in patients with brain injuries, was used, consisting of a modified PASAT task with numbers, words, and months of the year, according to Serino et al. (29) procedure.

The A treatment was performed, independently of the single tests’ impairment and was conducted by the psychologist by using conversation about the patient’s disease perception, his/her work, family, and hobbies, with the aim not to specifically exercise a cognitive ability, avoiding to treat depression or to have any behavioral or psychoanalytic approach. All the psychologists were trained by attending 10 consecutive training meetings with the psychologists of the coordinator center.

All the patients, after the neuropsychological examination at 1 year follow up, were considered as recovered, if the neuropsychological examination was normal, or still impaired, if not.

### Statistical analysis

Descriptive statistics of quantitative variables are expressed as median, 25th and 75th percentiles.

Due to low sample size in each group and to the not normally distributed variables under examination, the two groups were compared using Mann–Whitney statistic test for quantitative variables and Fisher Exact test for qualitative variables.

To assess the association between the difference in the number of pathological tests and the type of treatment a multiple linear regression model was fitted using the following covariates: EDSS change, mFIS, MSQoL, MADRS, number of relapse in the previous year, steroid consumption, sex, and age at T12.

All statistical analyses were performed using STATA/SE version 12.1 software (STATA/SE, 2011).

## Results

One hundred and fifty MS patients with the requested disease and treatment characteristics were submitted to neuropsychological evaluation, from April 2010 to December 2012. Of these, 109 were excluded; 89 because of they did not have the requested neuropsychological characteristics, 16 because they declined to participate (mainly for organization problems due to work/household reasons), and 45 were included. Four were not randomized to treatments, due to early drop out (for difficulties in accessing the MS Center in two cases and in maintaining the treatment due to work in the other two cases).

**Figure 1:**
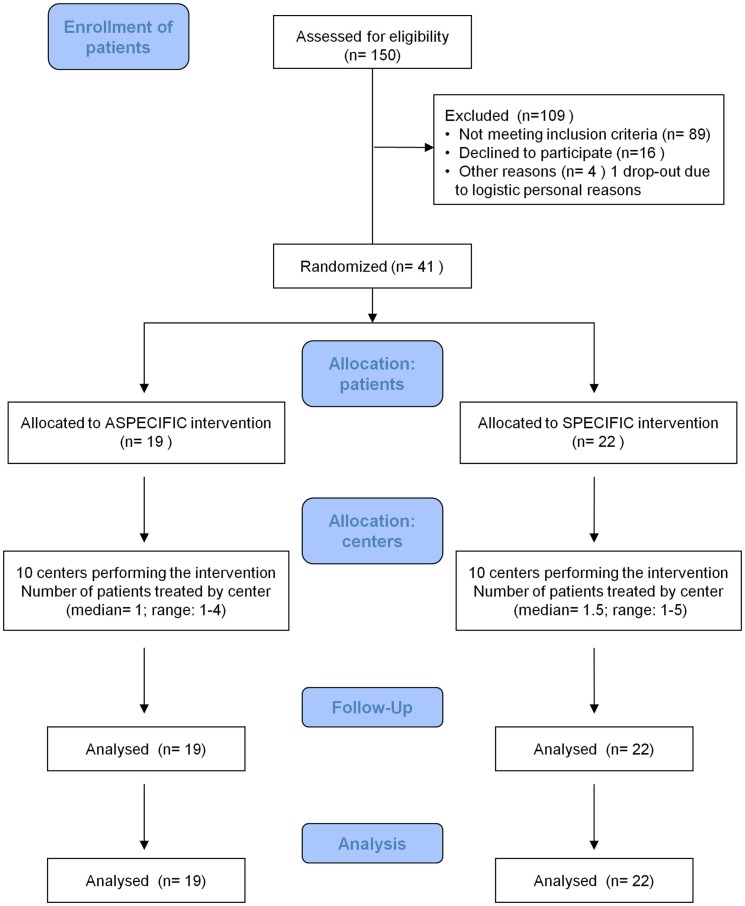
**Consort 2010 flow diagram**.

Forty one patients were randomly assigned to treatment A (19 patients) and treatment S (22 patients). Details are shown in Figure 1.

Patients’ groups were not significantly different for age, years of education, disease duration, relapse number in the previous year, EDSS, steroid consumption in the previous year (Table 1), and gender (males/females: 8/11 in group A and 9/13 in group S, n.s. Pearson χ^2^ test).

**Table 1:** **Characteristics of the study sample at baseline (T0)**.

	Treatment A (*n* = 19)			Treatment S (*n* = 22)			*p*-Value*
	25th %	Median	75th %	25th %	Median	75th %	
Age (years)	34	43	53	38	45	50	0.74
Years of education	8	13	14	8	9.5	15	0.47
Disease duration (months)	12	36	96	12	23.5	120	0.49
Relapses (previous year)	1	1	2	0	1	2	0.96
EDSS	1	2	3.5	2	2	3	0.78
Steroid (gr)	0	5	7	0	5	6	0.47

*Mann–Whitney test.

*A, aspecific; S, specific; EDSS, Expanded Disability Status Scale*.

Baseline neuropsychological evaluation did not show any statistically significant difference in the mean number of impaired tests between the two groups (2.21 ± 1.18 in A and 2.91 ± 1.48 in S group; n.s. Mann–Whitney test), demonstrating a generally moderate impairment in patients’ cognition. In addition, the mean raw score of tests at baseline was not significantly different between groups, except for SRT DR, which was marginally significantly lower in group S compared to group A. M Fis, MsQol, and MADRS sores were not different at follow up (Table 2).

**Table 2:** **Comparison of raw scores of neuropsychological tests at baseline (T0) between the two groups**.

Test	Treatment A (*n* = 19)			Treatment S (*n* = 22)			*p*-Value*
	25th %	Median	75th %	25th %	Median	75th %	
PASAT3″	25	35	40	27	35	43	0.61
PASAT2″	18	23	32	20	24.2	30	0.86
SPART10/36	14	17	23	13	14.5	21	0.33
SPARTDR	4	6	7	3	5	7	0.33
SRTLTS	29	34	46	24	30	44	0.31
SRTCLTR	21	24	40	17	21	36	0.15
SRTDR	6	8	9	6	6.5	8	0.044
SDMT	28	39	47	33	44	50	0.33
COWAP	23	30	37	25	34.5	40	0.15
COWAC	32	41	48	31	43.5	51	0.93
Stroop	13	20	30	19	20	29	0.74
MSQoL	130	167	186	138	167	201	0.68
MADRS	4	7	17	3	8	14	0.81
mFIS	6	26	46	14	29.5	47	0.62

*Mann–Whitney test.

*A, aspecific; S, specific; PASAT3″, PASAT2″, Paced Auditory Serial Addition Test; SPART10/36, 10/36 Spatial Recall Test for visuo-spatial learning – long-term retrieval; SPARTDR, Spatial Recall Test for visuo-spatial learning – delayed recall; SRTLTS, Selective Reminding Test – Long-Term storage; SRTCLTR, Selective Reminding Test – Consistent Long-Term Retrieval; SRTDR, Selective Reminding Test – delayed recall; SDMT, Symbol Digit Modalities Test; COWAP, Controlled Oral Words Association – Phoneme; COWAC, Controlled Oral Words Association – Category*.

*Stroop: MSQoL, Multiple Sclerosis Quality of Life; MADRS, Montgomery–Asberg Depression Rating Scale; mFIS, Modified Fatigue Impact Scale002E*.

The number of rehabilitative sessions performed in the first year, did not differ between groups (27.79 ± 5.48 in A and 26.32 ± 10.19 in S; n.s. Mann–Whitney test). In particular, S treatments were distributed as follows (denominators are the total number of patients assigned to S treatment): Memory in 16 cases (72%), Plan a Day in 11 (50%), and A/IP in 12 cases (55%), confirming the well known data that memory is the most frequently impaired cognitive domain in MS.

After rehabilitation, the mean number of impaired neuropsychological tests was found to be significantly lower in group S (3.53 ± 2.12 in A and 1.95 ± 2.97 in S; *p* < 0.01 Mann–Whitney test), demonstrating that patients submitted to treatment S improved after 1 year, whereas those ones submitted to the A treatment worsened. Furthermore, at T12 we found a statistically significant difference in the number of recovered patients; that is, patients with completely normal neuropsychological examination (1/19 for treatment A vs. 11/22 for treatment S, Pearson χ^2^ test *p* = 0.002).

The advantage of group S was confirmed also by analyzing the change (T12-T0) in the number of pathological tests between groups, which revealed a significant improvement in S (−0.95 ± 2.24) compared to A patients (1.32 ± 1.60, *p* = 0.0005 Mann–Whitney test).

Comparison of change (T12-T0) on raw score of neuropsychological tests between groups showed a significantly higher difference in group S for SRT DR and SPART 10/36 (Table 3), indicating that memory is the cognitive domain that most significantly changes after treatment S.

**Table 3:** **Comparison of changes in the neuropsychological tests raw scores at baseline (T0) and after rehabilitation (T12) between the two groups**.

Difference on raw scores T12–T0	Treatment A (*n* = 19)			Treatment S (*n* = 22)			*p*-Value*
	25th %	Median	75th %	25th %	Median	75th %	
ΔPASAT3	0	4	9	2	6	10	0.46
ΔPASAT2	0	3	8	0	8	10	0.42
ΔSPART10/36	−1	0	5	1	4	7	0.0395
ΔSPARTDR	−1	0	3	0	1	4	0.36
ΔSRTLTS	0	6	17	4	10	16	0.34
ΔSRTCLTR	−4	4	12	2	7.5	16	0.22
ΔSRTDR	−1	0	1	1	1.5	3	0.0076
ΔSDMT	0	1	5	1	3	7	0.24
ΔCOWAP	−2	1	4	−1	3	8	0.36
ΔCOWAC	−2	2	6	2	3.5	7	0.2
ΔStroop	−1	2	5	−1	2	7	0.96
ΔMSQoL	9	1	7	−12	0	9	0.98
ΔMADRS	−4	0	1	−3	−0.5	1	0.72
ΔmFIS	−9	−1	4	−8	−2.5	0	0.52

*Mann–Whitney test.

The within groups analysis of change (T12-T0) on raw scores in neuropsychological tests shows that patients treated with S treatment significantly improve in almost all the tests and in the fatigue scale, whereas patients treated with A significantly improve in PASAT 2″ and PASAT 3″ only (Table 4).

**Table 4:** **Comparison of change in neuropsychological tests raw scores at baseline (T0) and after rehabilitation (T12) within the two groups**.

	Treatment A (*n* = 19)		*p*-Value*	Treatment S (*n* = 22)		*p*-Value*
	T0 Median	T12 Median		T0 Median	T12 Median	
PASAT3	35	36	0.0014	35	44.5	0.002
PASAT2	23	30	0.0012	24.2	33.5	0.0070
SPART10/36	17	19	0.47	14.5	21.5	0.0008
SPARTDR	6	6	0.37	5	7	0.0352
SRTLTS	34	40	0.05	30	42	0.0004
SRTCLTR	24	28	0.13	21	31	0.0010
SRTDR	8	8	0.29	6.5	8	0.0007
SDMT	39	40	0.06	44	47.5	0.0004
COWAL	30	30	0.26	34.5	34.5	0.0456
COWAC	41	42	0.29	43.5	44.5	0.0260
Stroop	20	27	0.05	20	29	0.09
MSQoL	167	151	0.98	167	157.2	0.90
MADRS	7	8	0.81	8	6	0.23
mFIS	26	18	0.53	29.5	26	0.09

*Wilcoxon signed-rank test.

The multivariate regression analysis shows that the type of treatment is significantly associated with the difference in the number of impaired tests (*p* < 0.001). Specifically, patients treated with the S treatment have on average 2 impaired tests less than patients treated with A treatment, after adjusting for demographic, clinical and quality of life parameters (gender, age at T12, EDSS, number of relapses, steroid use, mFIS, MSQoL, and MADRS). Both in A and in S group we could not find any significant change in MSQoL, m Fis, and MADRS after 1 year.

Considering the specificity of the single S treatment (Plan a Day, Attention/IP, and Memory) used for the function treated, compared to the A approach, we also found a significantly higher improvement in group S for memory tests (SRT DR, SPART 10/36) and Attention/IP (SDMT), compared to group A (Table 5), showing that S training is better than A, particularly in treating memory and attention/IP functions, for which an increase ranging from four to fivefold was observed in tests’ scores. No statistically significant difference was found for executive function tests and Plan a day training.

**Table 5:** **Comparison of change in Executive function, Attention and Memory domain neuropsychological tests (raw scores T12-T0) within the A group and the three S treatments**.

	Treatment A			Treatment S			*p*-Value*
	25th %	Median	75th %	25th %	Median	75th %	
**Executive functions (19 vs. 11 patients)^a^**				**Plan a day**			
ΔCOWA C	−2	1	4	−1	7	14	0.32
ΔCOWA P	−2	2	6	−7	3	5	0.88
ΔStroop	−1	2	5	−1	1.5	10	0.84
**Attention (19 vs. 12 patients)^a^**				**Attention/IP**			
ΔPASAT3	0	4	9	1	9.5	15	0.21
ΔPASAT2	0	3	8	4	9	15	0.15
ΔSDMT	0	1	5	2.5	6.5	9	0.0263
**Memory (19 vs. 16 patients)^a^**				**Memory**			
ΔSPART10/36	−1	0	5	1	3.5	7.5	0.0394
ΔSPARTDR	−1	0	3	−0.5	1.5	4	0.31
ΔSRTLTS	0	6	17	4.5	10	17.5	0.29
ΔSRTCLTR	−4	4	12	3	7.5	15	0.18
ΔSRTDR	−1	0	1	0.5	1	2.5	0.03

*Mann–Whitney test.

*^a^Number of patients of the S group submitted to the domain specific treatment vs. the patients of the A group submitted to the A treatment*.

## Discussion

Based on previous research, we hypothesized that an intervention with specific exercises for single patient’s neuropsychological deficit would yield significantly larger cognitive benefits than aspecific psychological care. We found that MS patients engaged in a specific cognitive training significantly reduced the number of impaired neuropsychological tests and in general improved in tests’ scores. We observed no such improvements in the group assigned to an aspecific training. Importantly, although PASAT scores ameliorated after 1 year also in aspecific group – possibly due to a general effect on attention, the changes in tests’ performance after 1 year significantly favored the Specific group in 10 out of 11neuropsychological test. These differences and improvements are significant not only between groups but also within groups. Patients were well matched for both clinical and therapeutic characteristics, in order to ensure that the improvement was attributable to the type of the cognitive training only; in particular this trial is the first one, to our knowledge, in which a specific cognitive treatment is compared to an aspecific psychological intervention on relapsing-remitting MS patients all prescribed with the same immunomodulatory drug from the same time. Moreover, a multicenter study permits to replicate the same rehabilitative procedure in different centers. The effect of domain specific treatment is relevant, as at 1 year follow up, approximately 40% of patients submitted to the Specific treatment completely recovered compared to only 5% of patients in the Aspecific group, who, on the contrary, generally worsened. All the treated cognitive areas improved after specific exercises. This confirms our previous results on attention/information processing (12, 13) and also recent data from Chiaravalloti et al. (11) on memory.

Surprisingly, neither MSQoL nor MADRS (measures of quality of life and depression) were improved by treatment A, showing that an aspecific psychological intervention does not prove to be useful both on MS cognitive and on mood disorders.

Considering the high frequency of cognitive deficits in MS patients and their impact on patients’ activities, there is an urgent need to establish therapeutic interventions able to significantly alleviate these deficits. Despite cognitive rehabilitation being widely used in clinical practice, recent Cochrane revisions (9, 30) stated that, mainly due to methodological limitations of the currently available studies and subjects’ heterogeneity, at present there is a low level of evidence for the positive effects of neuropsychological rehabilitation in MS. Rehabilitation of selected cognitive domains (e.g., attention, EF, memory) has been associated with improved cognitive performance in the trained function in patients with MS (11, 12, 16, 17, 19), although often the training group was matched with no treatment group. In our study, a domain specific cognitive treatment was compared to an aspecific psychological care also in attention/IP and executive function deficits and the effects were found at 1 year. Our patients were all treated with interferon beta 1A 44 mcg three times/week no later than 6 months before randomization, the sample was well matched, with a moderate cognitive impairment, consistent with the disease type of the patients studied and other possibly inducing cognitive improvement other than the training may be ruled out. Furthermore, we can reasonably exclude that S patients could have improved due to a practice effect and not to learning of new strategies; in fact training procedures are very different from the tests’ tasks. In addition, these were used in alternate forms.

In conclusion, data show that relatively short periods of domain specific cognitive training can be helpful in ameliorating the trained function with effects persisting at 1 year and are superior to aspecific psychological interventions.

It is worth considering that the cognitive treatment was generally well tolerated and accepted, although in some centers, a practical difficulty in carrying out the complete rehabilitation program was observed: particularly a reduced compliance was reported for those cases where the rehabilitation had to be performed within the General Hospital, where most of the italian MS Centers are located, and not in a separate and possibly more easily accessible neuropsychological rehabilitation unit. Finally, the duration of the program could have had a role in reducing the size of the sample in our study; a shorter, but equally effective programs of cognitive rehabilitation could be more useful (31).

## Conflict of Interest Statement

The authors declare that the research was conducted in the absence of any commercial or financial relationships that could be construed as a potential conflict of interest.
